# Real-world efficacy and safety of pangenotypic direct-acting antivirals against hepatitis C virus infection in Taiwan

**DOI:** 10.1038/s41598-021-93095-x

**Published:** 2021-06-29

**Authors:** Kao-Chi Chang, Shui-Yi Tung, Kuo-Liang Wei, Chen-Heng Shen, Yung-Yu Hsieh, Wei-Ming Chen, Yi-Hsing Chen, Chun-Hsien Chen, Chi-Wei Yen, Huang-Wei Xu, Wei-Lin Tung, Chao-Hung Hung, Sheng-Nan Lu, Te-Sheng Chang

**Affiliations:** 1 Division of Hepatology and Gastroenterology, Department of Internal Medicine, Chang Gung Memorial Hospital, No. 6, Section West, Chiapu Road, Puzi, Chiayi 613 Taiwan, ROC; 2 Chang Gung University College of Medicine, Taoyuan, Taiwan, ROC

**Keywords:** Diseases, Gastroenterology

## Abstract

Clinical trials showed pangenotypic direct-acting antivirals’ (DAAs) excellent efficacy and safety when treating hepatitis C virus (HCV). Two pangenotypic regimens were examined, glecaprevir/pibrentasvir (GLE/PIB) and sofosbuvir/velpatasvir (SOF/VEL), in a real-world Taiwanese setting, including all HCV patients treated with GLE/PIB or SOF/VEL from August 2018 to April 2020. The primary endpoint was sustained virologic response 12 weeks after treatment cessation (SVR12), including adverse events (AEs). A total of 1,356 HCV patients received pangenotypic DAA treatment during the study: 742 and 614 received GLE/PIB and SOF/VEL, respectively. The rates of SVR12 for GLE/PIB and SOF/VEL were 710/718 (98.9%) and 581/584 (99.5%), respectively, by per-protocol analysis, and 710/742 (95.7%) and 581/614 (94.6%), respectively, by evaluable population analysis. Eleven (GLE/PIB: 8, SOF/VEL: 3) did not achieve SVR12. The most common AEs for GLE/PIB and SOF/VEL were pruritus (17.4% vs. 2.9%), abdominal discomfort (5.8% vs. 4.4%), dizziness (4.2% vs. 2%), and malaise (3.1% vs. 2.9%). Laboratory abnormalities were uncommon; only < 1% exhibited elevated total bilirubin or aminotransferase levels with both regimens. Five drug discontinuations occurred due to AEs (bilirubin elevation: 3; dermatological issues: 2). Pangenotypic DAAs GLE/PIB and SOF/VEL are effective and well tolerated, achieving high SVR12 rates for patients with all HCV genotypes.

## Introduction

Hepatitis C virus (HCV) infection is a major global public health problem and is associated with serious complications, including cirrhosis, hepatocellular carcinoma (HCC), and mortality. According to the global hepatitis report published by the World Health Organization, HCV infected 71 million people worldwide and caused ~ 400,000 deaths in 2015^1^. The goal of HCV infection treatment is to achieve a sustained virological response (SVR). An SVR is defined as the absence of detectable levels of HCV ribonucleic acid (RNA) in the blood at least 12 weeks after completing treatment. An SVR after treatment with direct-acting antivirals (DAAs), or interferon plus ribavirin, significantly decreases the complications of liver disease, extrahepatic manifestations, risk of HCC, and mortality^2–6^.

HCV is a minute enveloped single-stranded virus of 9,600-kb RNA belonging to the Flaviviridae family. There are seven known HCV genotypes with genome differences of more than 30% at the nucleotide level. Genotypes 1–6 are the most studied variants^7^. Based on the rapid progress in the treatment of HCV infection in recent years, interferon-free DAAs have proven to be effective and safe and have become the standard of care for HCV infection^8^. The choice of a suitable DAA regimen is complex initially, and the HCV genotype is one of the major factors determining an appropriate genotype-specific DAA. With the advent of pangenotypic DAA regimens, hepatitis C treatment has been simplified, and HCV genotyping is no longer an absolute requirement before initiating treatment^9,10^.

Glecaprevir/pibrentasvir (GLE/PIB) (100/40 mg, Maviret, Fournier Laboratories Ireland Limited Anngrove, Carrigtwohill, Cork, Ireland) is a fixed-dose combination (FDC) of two pangenotypic DAAs: GLE, a nonstructural viral protein 3/4A (NS3/4A) protease inhibitor, and PIB, a novel next-generation nonstructural viral protein 5A (NS5A) inhibitor with potent pangenotypic activity with or without compensated cirrhosis in patients aged ≥ 12 years^11–14^. Depending on the patient’s genotype, whether cirrhosis is present, and prior treatment experience, the recommended dose is three tablets (GLE 300 mg/PIB 120 mg) once daily for eight or 12 weeks^9^. Sofosbuvir/velpatasvir (SOF/VEL) (400/100 mg, Epclusa, Gilead Sciences Inc., Foster City, CA) is also an FDC of two pangenotypic DAAs: SOF, a liver-targeted pyrimidine nucleotide analogue that functions as a chain terminator and inhibits nonstructural viral protein 5B (NS5B) RNA-dependent RNA polymerase with pangenotypic potency, and VEL, a potent pangenotypic NS5A inhibitor. SOF/VEL is approved for chronic HCV genotypes 1 through 6, without ribavirin, in patients aged ≥ 6 years with or without compensated cirrhosis^15–19^. It is administered using a weight-based dosing regimen of ribavirin in patients with decompensated cirrhosis. The recommended dose is one tablet (SOF 400 mg/VEL 100 mg) once daily for 12 weeks^9^.

Previous clinical trials have demonstrated excellent efficacy and safety of both GLE/PIB and SOF/VEL in their respective patient cohorts. However, large-scale, real-world data involving these two pangenotypic DAAs are still limited. We evaluated the real-world efficacy and safety of GLE/PIB and SOF/VEL in a large cohort of patients with HCV infection of various genotypes in Taiwan.

## Methods

### Patients

In Taiwan, the National Health Insurance (NHI) has funded DAA treatment of patients with HCV infections since 2017. Between 2017 and 2018, this nationwide government-funded program enrolled only chronic HCV-infected patients with a hepatic fibrosis stage ≥ F3. However, in 2019, Taiwan’s NHI extended its coverage and approved treatment of all HCV viraemic patients regardless of the severity and duration of liver disease. GLE/PIB was approved by Taiwan’s Food and Drug Administration (TFDA) on January 19, 2018 and reimbursed by Taiwan’s NHI on August 1, 2018. SOF/VEL was approved by the TFDA on December 17, 2018 and reimbursed by Taiwan’s NHI on June 1, 2019.

Between August 2018 and April 2020, all patients aged ≥ 20 years with quantifiable serum HCV RNA (Cobas AmpliPrep/Cobas TaqMan HCV quantitative test v2.0, Roche Molecular Systems Inc., Pleasanton, CA, USA, lower limit of detection [LLOD]: 15 IU/mL across all HCV genotypes) who received GLE/PIB for 8 or 12 weeks or SOF/VEL for 12 weeks, with or without ribavirin, at Chiayi Chang Gung Memorial Hospital (CGMH) were included in the study.

This is a retrospective-prospective study approved by the Institutional Review Board of Chang Gung Medical Foundation on April 15, 2019 (IRB No.: 201900542B0) and was conducted in accordance with the principles of the Declaration of Helsinki and the International Conference on Harmonization for Good Clinical Practice. All patients gave their informed consent prior to their inclusion in the study.

### Study design

The following data were collected for all patients: basic demographics, prior treatment experience, prior history of HCC, presence of diabetes mellitus, hepatitis B virus (HBV) surface antigen, HCV RNA quantification, HCV genotype (Cobas HCV GT kit based on the Cobas 4800 system from Roche Diagnostics, Roche Molecular Systems Inc., Pleasanton, CA, USA), fibrosis index based on four factors (FIB-4), hemogram, serum albumin, total bilirubin, aspartate aminotransferase (AST), alanine aminotransferase (ALT), creatinine, estimated glomerular filtration rate (eGFR), and alpha-fetoprotein. Advancement of the liver disease was determined based on the FIB-4, acoustic radiation force impulse (ARFI) (Siemens Acuson S2000, Virtual Touch™ tissue quantification; Siemens), or the presence of portal hypertension based on image examinations.

The treatment regimen and duration were determined according to the current international guidelines for HCV infection and at the discretion of the treating physician. Patients with HCV genotypes 1–6 without liver decompensation received three oral GLE/PIB tablets with food once daily for eight or 12 weeks. When SOF/VEL was prescribed for HCV genotype 1–6 patients, the treatment duration was universally 12 weeks, and weight-based ribavirin (Robatrol, 200 mg capsule, Genovate Biotechnology Co. Ltd.) was added for patients with decompensated cirrhosis. All patients received follow-up for a minimum of 12 weeks after completing treatment.

### Assessment of efficacy

The primary efficacy endpoint was the SVR rate at 12 weeks after completing treatment (SVR12). The SVR12 rate was defined as the proportion of patients who had serum HCV RNA levels less than the LLOD at off-treatment week 12. The SVR12 rates in both the evaluable population (EP) (patients who received at least one dose of GLE/PIB or SOF/VEL) and per-protocol population (PP) (patients who received at least one dose of GLE/PIB or SOF/VEL, and whose HCV RNA data at post-therapy week 12 was available) were reported. The secondary efficacy endpoint was the rate of HCV RNA levels less than the LLOD at the end of treatment (EOT). Non-response was defined as detectable HCV RNA at the EOT. Relapse was defined as undetectable HCV RNA levels at the EOT, but detectable HCV RNA at off-treatment week 12.

### Assessment of safety

The payment regulations of Taiwan’s NHI stipulate that patients receiving DAA therapy must return to the hospital at least every four weeks during treatment and three months after completing treatment. Safety was evaluated using anamnesis, physical examination, and laboratory tests, and recorded in the CGMH electronic medical records database by physicians during every visit. Contraindicated drug-drug interactions (DDIs) between GLE/PIB or SOF/VEL and concomitant medications were assessed before and during the therapeutic course. In patients who experienced serious AEs or discontinued treatment, the causal relationship between the events and DAAs and the reasons for discontinuation were reviewed from the medical records. The following were considered serious treatment-related AEs: death; hepatic decompensation (variceal haemorrhage, ascites, hepatic encephalopathy, or prolonged prothrombin time > 3 s); grade 3 hyperbilirubinemia (total bilirubin levels more than 3 times above the upper limit of the normal (ULN) range); and HBV reactivation (presence of an HBV DNA level ≥ LLOD in patients with undetectable baseline HBV DNA or an increase in the HBV DNA level to ≥ 1 log_10_ IU/mL in patients with detectable baseline HBV DNA).

### Statistical analysis

Continuous data are expressed as means ± standard deviations or medians and ranges. Dichotomous data are expressed as numbers (percentages). Qualitative and quantitative differences between groups were analysed using the chi-square test for dichotomous variables and the Student’s t-test or Mann–Whitney U test for continuous variables, as appropriate. IBM SPSS Statistics for Windows, version 22.0 (IBM Corp., Armonk, N.Y., USA) was used for statistical analysis. Statistical significance was set as a two-tailed *P* value < 0.05.

## Results

### Patient characteristics

Between August 16, 2018, and April 22, 2020, a total of 1 356 patients with HCV viremia received pangenotypic DAA treatment at the CGMH. A total of 742 patients received GLE/PIB and 614 received SOF/VEL. Baseline patient characteristics for both the GLE/PIB and SOF/VEL groups were similar (Table 1). The greatest differences between the two groups were the use of ribavirin and treatment durations. The patients in the GLE/PIB group did not require ribavirin and most patients without cirrhosis underwent an 8-week treatment course, whereas all patients in the SOF/VEL group received 12 weeks of therapy, and ribavirin was required for patients with decompensated liver cirrhosis. The number of treatment-experienced patients in the GLE/PIB group (n = 96, 12.9%) was higher than that in the SOF/VEL group (n = 28, 4.5%). This is because GLE/PIB was approved by the TFDA and reimbursed by Taiwan’s NHI earlier than SOF/VEL. Thus, most patients who experienced failure of antiviral therapy had received GLE/PIB as soon as it started being reimbursed. Another significant difference was the higher baseline creatinine levels in the GLE/PIB group. Most patients with renal function impairment received GLE/PIB before the formal approval of SOF/VEL for the treatment of patients with chronic kidney disease.

**Table 1:** Baseline characteristics of patients.

	GLE/PIB	SOF/VEL	*p*-value
Number	742	614	
Age, year, mean ± SD	62.12 ± 12.95	63.31 ± 14.24	0.111
Sex, Male/Female	375/367	290/324	0.225
**Treatment experience (PR)**			< 0.001
Naïve/experienced	646/96	586/28	
**HBV coinfection**			0.181
Absent/present	676/66	546/68	
**Prior HCC history**			0.348
No/yes	714/28	583/31	
**Treatment duration, week**			< 0.001
8/12	622/120	0/614	
**Ribavirin usage**			< 0.001
No/yes	742/0	588/26	
**Diabetes mellitus**			< 0.001
No/yes	523/219	485/129	
**HCV RNA, IU/mL**			0.471
< 800,000/ ≥ 800,000	235/507	203/411	0.586
HCV RNA, IU/mL, median (range)	2,282,480 (18–40,230,995)	1,777,346 (51–34,466,974)	0.047
**HCV genotype**			Total
1a	20	16	36 (2.65%)
1b	138	248	386 (28.47%)
2	492	296	788 (58.11%)
3	12	4	16 (1.18%)
6	17	12	29 (2.14%)
Mixed	63	38	101 (7.45%)
FIB-4, > 3.25/ ≤ 3.25	160/582	143/471	0.447
Haemoglobin, g/dL, mean ± SD	13.67 ± 1.88	13.52 ± 1.89	0.138
White blood cell count, 10^9^ cells/L, mean ± SD	6.34 ± 1.93	6.52 ± 4.03	0.283
Platelet count, 10^9^ cells/L, mean ± SD	197.20 ± 63.17	198.93 ± 77.40	0.658
Albumin, g/dL, mean ± SD	4.28 ± 0.38	4.18 ± 0.50	< 0.001
Total bilirubin, mg/dL, mean ± SD	0.83 ± 0.35	0.98 ± 0.83	< 0.001
AST, U/L, mean ± SD	48.85 ± 38.94	51.37 ± 45.26	0.270
ALT, U/L, mean ± SD	60.72 ± 58.22	61.46 ± 67.80	0.829
Creatinine, mg/dL, mean ± SD	1.38 ± 1.92	0.96 ± 0.78	< 0.001
eGFR, mL/min/1.73 m^2^, mean ± SD	80.54 ± 30.71	84.94 ± 27.39	0.005
Alpha-fetoprotein, ng/mL, median (range)	3.1(0.5–3455.3)	3.3(0.7–16,509.5)	0.039

*ALT* alanine aminotransferase; *AST* aspartate aminotransferase; *eGFR* estimated glomerular filtration rate; *FIB-4* fibrosis index based on four factors; *GLE/PIB* glecaprevir/pibrentasvir; *HBV* hepatitis B virus; *HCC* hepatocellular carcinoma; *HCV* hepatitis C virus; *PR* peginterferon and ribavirin; *RNA* ribonucleic acid; *SD* standard deviation; *SOF/VEL* sofosbuvir/velpatasvir.

### Efficacy

**Figure 1:**
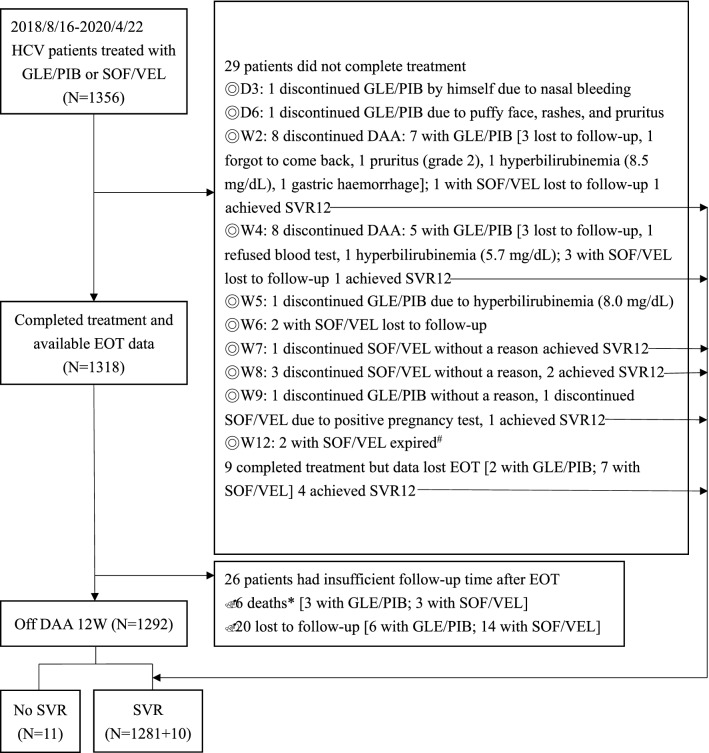
Flowchart of patient enrolment and withdrawal. # Both died at week 12 due to underlying decompensated liver cirrhosis and related complications. * One due to H1N1 influenza pneumonia 8 weeks off DAA, Two due to sepsis 1 week and 5 weeks, respectively, off DAA, one due to biliary tract infection with septic shock 10 weeks off DAA, one due to lung cancer or pneumonia 9 weeks off DAA, and **one** due to out-of-hospital cardiac arrest of unknown cause 6 weeks off DAA. *DAA* direct-acting antiviral; *SVR12* sustained virological response 12 weeks after treatment cessation; *GLE/PIB* glecaprevir/pibrentasvir; *SOF/VEL* sofosbuvir/velpatasvir; *HCV* hepatitis C virus; *EOT* end of treatment.

The flowchart of patient enrolment and withdrawal is presented in Fig. 1. Of the 1,356 patients who received pangenotypic DAA therapy, 1,318 completed the treatment course and had available EOT data. Of the 38 patients who did not complete the therapeutic protocol, 29 patients did not complete treatment and 9 patients completed treatment but without data at EOT. Ten of these 38 patients achieved SVR12.

A total of 29 patients were withdrawn from the pangenotypic DAA treatment: 16 in the GLE/PIB group and 13 in the SOF/VEL group. The reasons for patient withdrawal are shown in Table 2. At the EOT, 724 and 593 patients in the GLE/PIB and SOF/VEL groups, respectively, had undetectable serum HCV RNA levels. By EP analysis, the SVR12 rates were 710/742 (95.7%) and 581/614 (94.6%) in the GLE/PIB and SOF/VEL groups, respectively. By PP analysis, the SVR12 rates were 710/718 (98.9%) and 581/584 (99.5%) in the GLE/PIB and SOF/VEL groups, respectively (Table 3). Eleven patients did not attain SVR12: eight received GLE/PIB (relapses: 7, non-responder: 1) and three received SOF/VEL (non-responders: 3).

**Table 2:** Reasons for DAA withdrawal.

Reasons for withdrawal	GLE/PIB (N = 16)	SOF/VEL (N = 13)
Lost to follow-up	7	10
Forget to come back	1	
Refuse to check blood	1	
Bilirubin elevation	3	
Skin itching/rashes/puffy face	2	
Nasal bleeding	1	
Gastric haemorrhage	1	
Death		2
Positive pregnancy test		1

*DAA* direct-acting antiviral; *GLE/PIB* glecaprevir/pibrentasvir; *SOF/VEL* sofosbuvir/velpatasvir.

**Table 3:** Virologic responses.

HCV RNA < LLOD	Patient (N = 742)	Patient (N = 614)	*p* value
	GLE/PIB, n /N (%)	SOF/VEL, n /N (%)	
**End of treatment**			0.363
ETR (PP)	724/727 (99.6)	593/596 (99.5)	
Lost to follow-up	15	18	
**After treatment**			
SVR12 (EP)	710/742 (95.7)	581/614 (94.6)	0.228
SVR12 (PP)	710/718 (98.9)	581/584 (99.5)	0.228
**Reason for non-SVR12, n**			
Relapse	7	0	
Nonresponse	1	3	
Lost to follow-up	24	30	

*EP* evaluable population; *GLE/PIB* glecaprevir/pibrentasvir; *HCV* hepatitis C virus; *LLOD* lower limit of detection; *PP* per-protocol population; *RNA* ribonucleic acid; *SOF/VEL* sofosbuvir/velpatasvir; *SVR12* sustained virological response 12 weeks after treatment cessation.

The baseline characteristics of the 11 patients who did not attain SVR12 are summarised in the Supporting Information Table S1. The majority of these patients were treatment-naïve, and only one 77-year-old man with HCV genotype 2 without advanced liver fibrosis had prior treatment experience. All seven relapsers were treated with GLE/PIB, and only one had advanced liver fibrosis with a FIB-4 score of 3.87.

**Figure 2:**
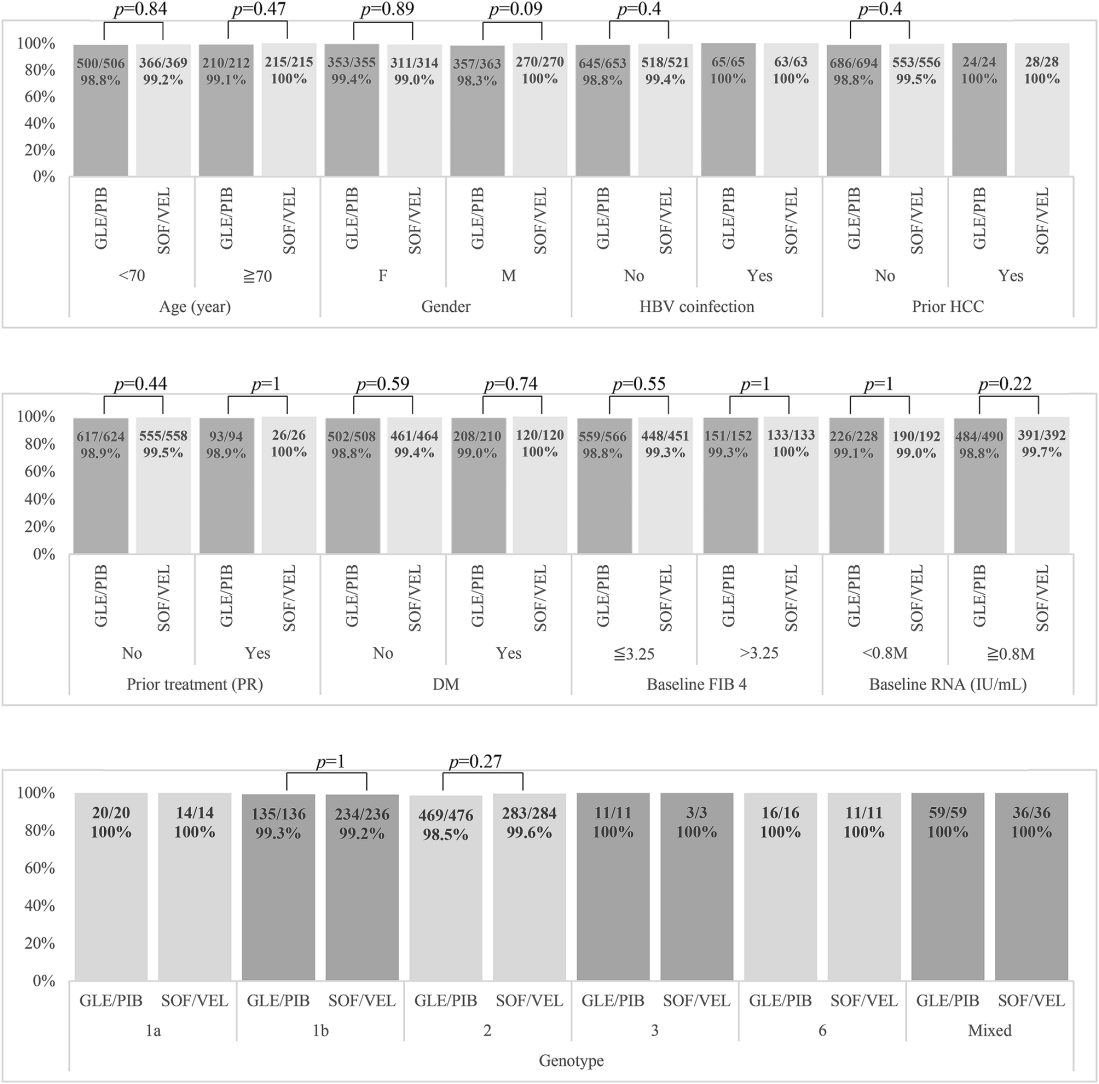
Subgroup analyses of SVR12 (per-protocol set). *FIB-4* fibrosis index based on four factors; *GLE/PIB* glecaprevir/pibrentasvir; *SOF/VEL* sofosbuvir/velpatasvir; *HBV* hepatitis B virus; *HCC* hepatocellular carcinoma; *RNA* ribonucleic acid; *SVR12* sustained virological response 12 weeks after treatment cessation.

Subgroup analyses for SVR12 rates by patient characteristics are shown in Fig. 2. The SVR12 rates were excellent (98.3–100%, PP analysis) and the efficacies were similar regardless of age, sex, HBV coinfection, prior HCC, prior treatment experience, diabetes mellitus, baseline advancement of the liver disease, baseline HCV RNA level and HCV genotype (*p* > 0.05).

### Safety outcomes

Of the 1356 patients, 1327 (97.8%) completed the therapeutic course. Two deaths occurred during week 12 of SOF/VEL treatment: a 76-year-old woman died due to underlying decompensated liver cirrhosis with progression and a 73-year-old man due to spontaneous bacterial peritonitis with septic shock. Six deaths occurred in the off-DAA treatment group during the 12-week follow-up. The causes of death were H1N1 influenza pneumonia, septic shock with metabolic acidosis, biliary tract infection with septic shock, lung cancer with respiratory failure, out-of-hospital cardiac arrest of unknown cause, and severe sepsis. None of the deaths was attributable to the DAA treatment. The most common AEs, comprising > 3% of the total patients in the GLE/PIB and SOF/VEL groups, were as follows: pruritus (17.4% vs. 2.9%), abdominal discomfort (5.8% vs. 4.4%), dizziness (4.2% vs. 2%), fatigue (3.1% vs. 2.9%), and elevation of total bilirubin in 1.5–3 × ULN (5.3% vs. 2.9%) (Table 4). Only five drug discontinuations resulted from AEs (bilirubin elevation: 3, dermatological: 2, in the GLE/PIB group).

**Table 4:** Safety summary.

Event, n (%)	GLE/PIB (N = 742)	SOF/VEL (N = 614)	Total (N = 1356)
**Adverse events (> 3%)**			
Skin itching	129 (17.4)	18 (2.9)	147 (10.8)
Abdominal discomfort	43 (5.8)	27 (4.4)	70 (5.2)
Dizziness	31 (4.2)	12 (2)	43 (3.2)
Malaise	23 (3.1)	18 (2.9)	41 (3)
**Laboratory adverse event, n (%)**			
Total bilirubin elevation			
1.5–3 × ULN	39 (5.3)	18 (2.9)	57 (4.2)
> 3 × ULN	4 (0.5)	6 (1)	10 (0.7)
AST elevation			
3–5 × ULN	3 (0.4)	9 (1.5)	12 (0.9)
> 5 × ULN	3 (0.4)	1 (0.2)	4 (0.3)
ALT elevation			
3–5 × ULN	6 (0.8)	4 (0.7)	10 (0.7)
> 5 × ULN	7 (0.9)	2 (0.3)	9 (0.7)

*ALT* alanine aminotransferase; *AST* aspartate aminotransferase; *GLE/PIB* glecaprevir/pibrentasvir; *SOF/VEL* sofosbuvir/velpatasvir; *ULN* upper limit of normal.

## Discussion

With the rapid development and evolution of anti-HCV drugs, pangenotypic DAAs have gradually replaced genotype-specific DAAs as the first-line therapy^9,10^. Pangenotypic DAAs may prevent physician errors associated with unfamiliarity with genotype-specific DAAs and broaden the number of eligible patients by reducing the need for genotyping. Our results confirmed that both GLE/PIB and SOF/VEL can serve as appropriate pangenotypic DAA regimens for the treatment of HCV in general practice. In this real-world setting involving a south Taiwanese cohort, our study revealed that the overall SVR12 rates in the GLE/PIB and SOF/VEL groups were 95.7% and 94.6%, respectively by EP analysis and 98.9% and 99.5%, respectively by PP analysis. This therapeutic efficacy was similar to those reported in previous clinical trials, meta-analyses, and real-world reports for GLE/PIB^11–13,20–22^ and SOF/VEL^15–18,23–25^. The real-world data currently available mainly involve the effects of a single drug. Large-scale, real-world, general population cohort studies comparing these two pangenotypic DAAs are limited. The government of Taiwan was the first in Asia to vigorously promote the treatment of HCV and to reimburse these two pangenotypic DAA regimens without restriction. We evaluated the real-world efficacy of both GLE/PIB and SOF/VEL for HCV treatment.

In the subgroup analyses, we evaluated the potential factors associated with SVR12 rates. Both the GLE/PIB and SOF/VEL groups exhibited excellent SVR12 rates regardless of baseline patient characteristics. This result was comparable to previous pooled analyses and other real-world reports^20–25^.

Regarding tolerability and safety, 726/742 (97.84%) and 601/614 (97.88%) of patients in the GLE/PIB and SOF/VEL groups, respectively, completed the treatment courses. Five patients in the GLE/PIB group did not complete the treatment due to reasons which may be related to the therapy: three due to bilirubin elevation and two due to pruritus. Two deaths, which were not related to the treatment, occurred in the SOF/VEL group during the therapeutic course. The most common AE in the GLE/PIB group was pruritus, and the proportion of patients with pruritus in our study (17.4%) was higher than that in other real-world studies (4.7–7.8%)^12,20,21^. This may be related to more chronic kidney disease patients, with higher baseline creatinine levels (1.38 ± 1.92 mg/dL), in the GLE/PIB group. In subjects with normal renal function, GLE/PIB is mostly eliminated through the faecal-biliary route and less than 1% of the drugs are recovered in the urine^26,27^. However, with decreasing renal function, increased GLE/PIB plasma exposure (up to 46–56%) has been reported^28^. Previous studies also showed higher pruritus rates (12–30.5%) in patients with severe renal impairment^29–31^. Although the pruritus rate was higher in our study, only two patients discontinued treatment for this reason. The most common AE in our SOF/VEL group was abdominal discomfort (4.4%), which was comparable to other studies^24^. Overall, this study confirmed that GLE/PIB and SOF/VEL are both well-tolerated and safe for patients with chronic HCV infection in Taiwan.

There were some limitations to this study. First, genotypes 4 and 5 are rare in Taiwan, so the pangenotypic therapeutic effect of GLE/PIB and SOF/VEL for these two genotypes was not studied. Second, the majority of data for this retrospective study was collected from electronic medical records; therefore, there were potential underreporting biases for mild to moderate AEs. Third, the HCV genotypes of the seven relapsers in the GLE/PIB group were not re-checked; therefore, the possibility of repeated infection cannot be excluded. Fourth, since the CGMH is one of the referral centres of southern Taiwan, therapeutic data for these two pangenotypic DAAs from local hospitals or private clinics are needed for further study.

In conclusion, the pangenotypic DAAs, GLE/PIB and SOF/VEL are well tolerated and show excellent SVR12 rates for patients infected with all genotypes of the HCV in real-world practice in Taiwan.

## Supplementary Information

Supplementary Table S1.

## Data Availability

Due to legal restrictions imposed by the government of Taiwan in relation to the “Personal Information Protection Act”, data cannot be made publicly available. Requests for data can be sent as a formal proposal to the corresponding author.
