# Supplementary material for: Real-world efficacy and safety of pangenotypic direct-acting antivirals against hepatitis C virus infection in Taiwan
Source: Sci Rep. 2021 Jun 29;11:13543. doi: 10.1038/s41598-021-93095-x (PMC8241842; doi:10.1038/s41598-021-93095-x)
Supplement: Supplementary file 1 — Supplementary Table S1. [file 41598_2021_93095_MOESM1_ESM.docx]

**Table S1. Baseline characteristics of 11 patients without achieving SVR12**

|  | Case 1 | Case 2 | Case 3 | Case 4 | Case 5 | Case 6 | Case 7 | Case 8 | Case 9 | Case 10 | Case 11 |
| --- | --- | --- | --- | --- | --- | --- | --- | --- | --- | --- | --- |
| Type of SVR12 failure | Non-responder | Relapse | Relapse | Relapse | Relapse | Relapse | Relapse | Relapse | Non-responder | Non-responder | Non-responder |
| DAA type | GLE/PIB | GLE/PIB | GLE/PIB | GLE/PIB | GLE/PIB | GLE/PIB | GLE/PIB | GLE/PIB | SOF/VEL | SOF/VEL | SOF/VEL |
| Ribavirin usage | No | No | No | No | No | No | No | No | No | No | No |
| Prior treatment | Naïve | Naïve | Naïve | Naïve | Naïve | Naïve | Yes | Naïve | Naïve | Naïve | Naïve |
| Gender | Female | Male | Male | Male | Male | Male | Male | Female | Female | Female | Female |
| Age, year | 68 | 62 | 34 | 53 | 76 | 62 | 77 | 67 | 36 | 62 | 40 |
| BMI, kg/m^2^ | 19 | 22.3 | 22.3 | 24.6 | 27.7 | 21.9 | 25.2 | 28.7 | 20.9 | 21.6 | 33.4 |
| DM | No | No | No | Yes | No | No | Yes | No | No | No | No |
| HBV infection | No | No | No | No | No | No | No | No | No | No | No |
| Prior HCC | No | No | No | No | No | No | No | No | No | No | No |
| Cirrhosis | Yes | No | No | No | No | No | No | No | No | No | No |
| FIB-4 | 3.19 | 1.84 | 0.42 | 1.15 | 3.08 | 1.20 | 1.59 | 3.87 | 0.67 | 1.48 | 1.09 |
| Genotype | 2 | 2 | 2 | 2 | 2 | 2 | 2 | 1b | 1b | 1b | 2 |
| HCVRNA, IU/mL | 862,135 | 2,289,421 | 16,446,639 | 3,448,930 | 605,583 | 22,352,770 | 13,781,893 | 98,883 | 22,047 | 1,293,629 | 335,076 |
| AST, U/L | 26 | 30 | 15 | 34 | 28 | 21 | 28 | 49 | 21 | 20 | 70 |
| ALT, U/L | 20 | 21 | 16 | 57 | 18 | 24 | 30 | 40 | 22 | 14 | 124 |
| Total bilirubin, mg/dL | 0.3 | 0.6 | 0.7 | 1 | 1.4 | 0.8 | 0.6 | 0.9 | 0.6 | 0.9 | 0.4 |
| Direct Bilirubin, mg/dL | 0.1 | 0.2 | 0.1 | 0.1 | 0.3 | 0.1 | 0.1 | 0.1 | 0.1 | 0.1 | 0.1 |
| Creatinine, mg/dL | 11.15 | 0.9 | 0.87 | 0.7 | 0.89 | 0.99 | 0.84 | 0.88 | 0.57 | 0.91 | 0.53 |
| eGFR, mL/min/1.73m^2^ | 3.63 | 90.88 | 106.76 | 125.38 | 88.33 | 81.41 | 94.18 | 68.12 | 127.56 | 66.58 | 135.8 |
| Albumin, g/dL | 4.2 | 4.4 | 4.7 | 4.8 | 4.6 | 4.5 | 4.4 | 4.4 | 4 | 4.3 | 4.5 |
| WBC, 1000/uL | 2.7 | 4.2 | 6.6 | 5.6 | 5.6 | 4 | 11.2 | 6.8 | 5.2 | 4.9 | 5.3 |
| Hgb, g/dL | 7.9 | 15.1 | 14.1 | 16.2 | 9.3 | 16.4 | 15.6 | 14.7 | 12.5 | 12.9 | 13.1 |
| PLT, 1000/uL | 124 | 220 | 307 | 207 | 163 | 222 | 247 | 134 | 241 | 224 | 230 |
| INR | 1.01 | 0.99 | 1.08 | 1.04 | 1.04 | 1 | 0.98 | 1.09 | 1.02 | 1.02 | 1.06 |

ALT: alanine aminotransferase; AST: aspartate aminotransferase; BMI: body mass index; DAA: direct-acting antiviral; eGFR: estimated glomerular filtration rate; FIB-4: fibrosis index based on four factors; GLE/PIB: glecaprevir/pibrentasvir; HBV: hepatitis B virus; HCC: hepatocellular carcinoma; HCV: hepatitis C virus; Hgb: Haemoglobin; INR: International normalized ratio; PLT: Platelet count; RNA: ribonucleic acid; SD: standard deviation; SOF/VEL: sofosbuvir/velpatasvir; SVR12: sustained virological response 12 weeks after treatment cessation; WBC: White blood cell count.
